# extendGAN+: Transferable Data Augmentation Framework Using WGAN-GP for Data-Driven Indoor Localisation Model

**DOI:** 10.3390/s23094402

**Published:** 2023-04-30

**Authors:** Seanglidet Yean, Wayne Goh, Bu-Sung Lee, Hong Lye Oh

**Affiliations:** 1 Singtel Cognitive and Artificial Intelligence Lab (SCALE@NTU), Nanyang Technological University, 50 Nanyang Avenue, Singapore 639798, Singapore; 2 School of Computer Science and Engineering, Nanyang Technological University, 50 Nanyang Avenue, Singapore 639798, Singapore

**Keywords:** indoor localisation, generative adversarial networks (GANs), convolutional neural network, transfer learning, received signal strength

## Abstract

For indoor localisation, a challenge in data-driven localisation is to ensure sufficient data to train the prediction model to produce a good accuracy. However, for WiFi-based data collection, human effort is still required to capture a large amount of data as the representation Received Signal Strength (RSS) could easily be affected by obstacles and other factors. In this paper, we propose an extendGAN+ pipeline that leverages up-sampling with the Dirichlet distribution to improve location prediction accuracy with small sample sizes, applies transferred WGAN-GP for synthetic data generation, and ensures data quality with a filtering module. The results highlight the effectiveness of the proposed data augmentation method not only by localisation performance but also showcase the variety of RSS patterns it could produce. Benchmarking against the baseline methods such as fingerprint, random forest, and its base dataset with localisation models, extendGAN+ shows improvements of up to 23.47%, 25.35%, and 18.88% respectively. Furthermore, compared to existing GAN+ methods, it reduces training time by a factor of four due to transfer learning and improves performance by 10.13%.

## 1. Introduction

With the rise of multi-floor developments, urbanisation, and smart cities increasing the living space and building complexity, indoor localisation and navigation has played a significant role in assisting users to their destination indoors. Its application has been deployed in commercial buildings [1,2], carparks [3], the health service [4], public transport [5,6], etc.

Indoor localisation has remained an active research area over the past few years. There has been a shift from using extra hardware to the hybrid techniques of the existing wireless technologies and data-driven machine learning, in which the fingerprint approach, with the WiFi-received signal strength (RSS), has high adoption rate for the indoor positioning system because of its effectiveness, simplicity, and minimal requirements for additional setup or hardware as it utilises the available pre-installed WiFi access points (APs). However, the RSS varies due to obstacles, multi-path phenomena, and fading effects, resulting in arbitrary fluctuations. Hence, to achieve the desired localisation accuracy, the machine learning approach [1,7,8] and deep learning approach [9] were explored. As a result, the deep learning approach has shown potential in learning the complex features from the training data, requires less effort in feature engineering, and achieves higher accuracy [10,11]. In summary, the desired positioning accuracy could be achieved using the deep learning model when the RSS shadowing and fading challenges, that affect the correction of RSS value and its node location, are addressed [12].

Although adopting the neural network prediction model means benefiting from its ability to learn without a well-constructed features/domain knowledge and to tolerate fault or corruption in the network, selecting the optimal architecture plays an important role improving performance of the model. For indoor application, the RSS-based model architecture started with the multi-layer perceptron [9] and rapidly adopted targeted architectures to extract relevant features by the feature reconstruction autoencoder [13] and/or spatially-aware convolution neural network (CNN) [14]. The CNN model has been shown to be an effective model for the RSS-based localisation application since it leverages the relationship of the wireless heatmap and the corresponding location. The study [15] took a step forward to incorporate time-dependent factors by inputting time-series RSS to the CNN model in order to address the randomness and noise caused by the RSS fluctuation issue. However, the CNN architecture was suggested based on empirical considerations. Thereupon, there is research interest to develop strategies for the model selection. It is seen in the adoption of well-performed CNN architecture. For instance, Ref. [16] introduces a VGG-block-building framework via heuristic hyperparameter search to optimise the the performance using CVTuner. In this study, various techniques ranging from the traditional machine learning method to the use of a CNN-based model for feature extraction to benchmark the performance.

A known drawback of training deep learning models is the prerequisite of sufficient good quality data, the pivotal component. For indoor localisation using RSS data, the site visit and data collection is essential. In other words, the larger the site, the more effort required to collect data for the premise, which is time consuming and labor-intensive [17,18,19]. For instance, Rashmi et al. reported having to collect at least 15 hours’ worth of data over a span of 7 days for the training of a CNN model [20]. To mitigate the data challenge, synthetic data are being generated to train data. Data augmentation methods for indoor localisation range from sampling with a defined distribution [2,20] to training a data-driven Generative Adversarial Network [21]. Although data aggregation using a defined distribution evidently improves the performance of the prediction model, there is a question of which distribution models the fluctuating nature of RSS and its environment. Therefore, more focus has been on the data-driven approach. In particular, a conditional GAN was introduced to perform data augmentation the targeted area with the received signal strength and its cell-ID location as input [22]. Even though the one-for-all augmenter learns the complex structure by using one-hot encoded location as the GAN’s auxiliary data, the training could become a complex one as the reference point grows from the reported 25 reference points. Wafa et al. took similar approach to generate data for the entire out (a one-for-all augmenter) and proposed the Selective Generative Adversarial Network with DNN model (Selective-SS-GAN) to predict pseudo-labelling at the unseen location. Meanwhile, AF-DCGAN trained a GANs per location, aiming to learn the data distribution for each unique location [23]. However, instead of RSS data, the method is used to generate additional amplitude feature maps of the Channel State Information (CSI) which requires additional hardware modification. Nonetheless, the method proposed for the CSI amplitude may not be applicable for RSS data as RSS characterises only the coarse value at the receiver and displays high variability. In summary, the exploration of data augmentation is crucial to the localisation performance where data-driven approach GANs show potential in augmenting better quality data. On the other hand, more generated data do not always improve the performance. The localisation’s performance in [22] stalemates when the generated data exceed an optimal point. Albeit proposing an augmentation method, the recommended amount of synthetic data were not addressed.

Addressing the aforementioned challenges in constructing the RSS fingerprint with synthetic data for the localisation model, the main contributions of this paper are the following:

- Introduce an end-to-end recommendation to improve the data-driven localisation model’s performance by proposing a data augmentation pipeline and residual-network adaptation for the feature extraction of the localisation model.
- Propose an extendGAN+ pipeline to generate synthetic data for the localisation model even with an extremely small training dataset. The approach leverage on the combination of up-sampling with the Dirichlet distribution at the location with below-threshold data points, transferred WGAN-GP and a filtering module for quality control. The use of transfer learning was to reduce the training time and computational resources while the WGAN-GP model was organically trained once at the location with maximum data point. In addition to the proposed method, we provide a practical recommendation to set the amount of the augmented data as more generated data do not always improve the performance.
- Conduct experiments with the publicly accessible dataset (UJIndoorLoc [24]) and self-collected data at the building complex to evaluate the proposed method to provide better insights of the difference in data quality and localisation performance. The state-of-the-art network architectures for indoor localisation were used for the additional performance evaluation.

## 2. Methodology

### 2.1. Overview

**Figure 1:**
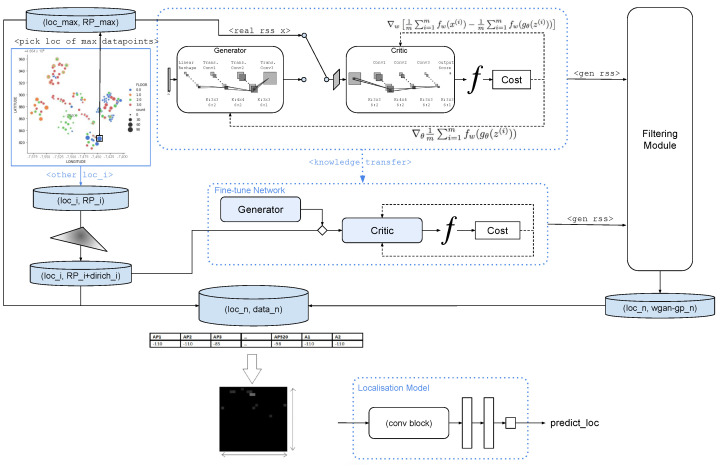
extendGAN+ Overview.

In this section, we propose the transferable workflow that includes data augmentation and localisation models (Figure 1).

The extendGAN+ framework leverage on the WGAN-GP to create synthetic RSS data as a WGAN-GP model is trained as per unique location. Firstly, the training dataset is sorted for the unique location with most data points ($loc_{max}$ where $data\left(RP_{max}\right)=max\left(data\left(RP_{i}\right)\right)$) and set data threshold ($d_{max}∼data\left(RP_{max}\right)$). Upon selecting $loc_{max}$, the WGAN-GP model is trained from scratch to produce augmented data. The work of  [22] has shown that the amount of generated data has an impact on the localisation accuracy, peaked and saturated. The 1:1 ratio is chosen according to our empirical study on the impact of generated data; hence, we are generating RSS in $d_{max}$ amount using the trained WGAN-GP. For another unique location ($loc_{i}$) with data point ($d_{i}$), where $loc_{i}\neq loc_{max}$, the Dirichlet data aggregation method is used to up-sample $d_{i}$ to the size of $d_{max}$. Subsequently, WGAN-GP model at $loc_{max}$ is transferred to $loc_{i}$ to generate augmented data of the above mentioned 1:1 ratio. In summary, at $loc_{i}$, we obtain the total data composition of original data ($d_{i}$), Dirichlet up-sampling data ($d_{i}^{dirichlet}=d_{max}=d_{i}$) and WGAN-GP-generated data ($d_{i}^{WGAN-GP}=d_{max}$). It is to be noted that the data used for augmentation are the training dataset, while keeping the test dataset untouched. Subsequently, the localisation model could be trained with the new training dataset.

### 2.2. Data Augmentation–extendGAN+

For data-driven indoor localisation, the RSS fingerprint is crucial in determining the location. However, the fingerprint varies due to disruptions such as the non-line-of-sight (NLOS) effect. More data collection sessions are needed at different times of days or weeks, in addition to being taking in various changing environment scenarios such as crowd size and environment. It mitigates the representation challenge, albeit at the cost of inefficiency in time and labour. Thus, data-augmentation methods were introduced.

In the previous study [25], we proposed GAN+ data augmentation using the GAN+ framework, which combines the Dirichlet and GAN augmentation techniques, to generate augmented RSS achieving improved performance even with a small training database. The augmentation was applied to one location at a time by aggregating data with Dirichlet distribution and train the GAN model. The synthetic data were then filtered to remove outliers. Although obtaining a variation of RSS representation, training a GAN model per location is time inefficient, especially when scaling up the testbed. Moreover, each location contains a varying number of data points. Thus, setting a static parameter does not address in-label imbalancing. Therefore, in this study, we propose an improved GAN+ (extendGAN+) framework with a localisation model.

### 2.3. GAN to WGAN-GP

Among the other generative models, Generating Adversarial Networks (also known as GANs) represent a state-of-the-art deep learning framework which does not involve the maximum likelihood estimation, and the generator is trained without having seen the real data. There have been a variety of applications using GANs and their variations in numerous fields such as the medical field [26], human face images [27], maps [28], etc. [29,30]. However, GANs suffer their own setbacks such as non-convergence and mode collapsing. The vanishing gradient, resulting in non-convergence, refers to the situation where the gradient update of the generator’s weight is close to zero, such that its weight would not be updated effectively, while mode collapsing focuses on the produced output to be of random yield rather than specific to the target output.

Mitigating the mentioned challenges, the Wasserstein Generative Adversarial Network with Gradient Penalty (WGAN-GP) presented an effective solution by incorporating Wasserstein distance to the critics loss function [31,32]. The Wasserstein distance measures the distance between two probability distributions by calculating the minimum cost of transporting mass in converting from one data distribution to another (Equation (1)). Furthermore, the Wasserstein distance is derived in Equation (2) where *f* satisfies constraint ($\left|f\left(x_{1}\right)-f\left(x_{2}\right)\right|\leq \left|x_{1}-x_{2}\right|$) to be a 1-Lipschitz function and sup refers to the supremum of a set. To enforce the 1-Lipschitz function, the gradient penalty was used such that gradient of the critics would have the unit norm (with $\lambda E_{\hat{x}∼P\hat{x}}\left[{\left({∥{▽}_{\hat{x}}f\left(\hat{x}\right)∥}_{2}-1\right)}^{2}\right]$ in Equation (3)).

(1) $$W\left(P_{r},P_{g}\right)=\underset{\gamma \in \Pi (P_{r},P_{g})}{inf}E_{\left(x,y∼\gamma \right)}\left[∥x-y∥\right]$$

(2) $$W\left(P_{r},P_{g}\right)=\underset{{∥f∥}_{L}\leq 1}{sup}E_{x∼P_{r}}\left[f(x)\right]-E_{x∼P_{g}}\left[f(x)\right]$$

(3) $$L_{critic}=E_{\tilde{x}∼Pg}\left[f(\tilde{x}\right)]-E_{x∼Pr}\left[f(x\right)]+\lambda E_{\hat{x}∼P\hat{x}}\left[{\left({∥{▽}_{\hat{x}}f\left(\hat{x}\right)∥}_{2}-1\right)}^{2}\right]$$

With the change of loss function to the continuous metric, WGAN-GP could quantify the performance better by focusing on the image quality rather than the binary classification for whether the generator network could fool the discriminator network with the Sigmoid function. As a result, WGAN-GP yields the training stability with no sign of mode-collapsing and the critics can still learn when the generator performs well by the change of loss function. Moreover, the gradient penalty reduces the efforts to perform hyper-parameter tuning, as compared to WGAN without gradient penalty having to set weight-clipping parameter *c*.

### 2.4. Upsampling with Dirichlet Data Aggregation

To compensate for the imbalance of training data towards the location of the majority of data points, upsampling is adopted to synthetically generate data and add bias into the dataset. As RSS variations in nature are highly affected by obstacles and the multipath and fading effects, it requires more data collection effort to capture the fingerprint representation of the location. Thus, upsampling RSS data is the right fit to prepare the data for the WGAN-GP model, compensating for the lack of data at certain location. In fact, the upsampling process for RSS data is commonly performed via permutation of the fingerprints by repetition or generating random numbers from a predefined distribution, such as uniform or normal distributions, using existing data.

In this study, we adopted the Dirichlet distribution to upsample data for the locations that lack data to train with WGAN-GP ($d_{i}<d_{max}$). The Dirichlet distribution is commonly used in the area of text mining and text network analysis to fit a topic model as it allows a mixture of topics and words to overlap (rather than being repeated in discrete group). Differing from uniform or normal distributions, the Dirichlet distribution provides versatility, which characterises the random variability, and is able to govern the shape of the distribution by its nature being a multivariate probability distribution. A Dirichlet distribution [33], represented by a vector $\vec{\alpha }$, has the probability density given by Equation (4).

(4) $$\rho \left(\vec{x}\right)=Z\prod_{i}x_{i}^{\alpha_{i}-1}$$

where random variable $x_{i}$ is distributed according to the Dirichlet distribution Dir(α) ($x_{i}∼Dir\left(\alpha \right)$) if density function $\rho (\vec{x})$ holds. $\alpha_{i}=\left\{\alpha_{1},\alpha_{2},...\alpha_{n}\right\}>0$ is a vector that holds the parameter of the distribution while *Z* is the generalised multinomial coefficient and *n* is the number of samples.

Therefore, in this study, instead of augmenting each fingerprint/record (by row) with random numbers generated from a pre-defined uniform or normal distribution, we use a Dirichlet-distributed random variable for **each access point (by column)**. The Dirichlet augmentation scheme requires at least 2 records from a given location (let the number of records at the unique location be *N*) (shown in Algorithm 1). Additionally, let the total number of access points be *t* and the number of datapoints in a unique location be *N*. In this scheme, new RSS fingerprints are generated by finding the weighted sum of the RSSI based on the individual APs. In other words, assigning random weight ($W=[W^{AP1},W^{AP2},...,W^{AP_{t}}]$) to each of these *N* records consisting of *t* measurements, such that the weights sum up to 1. The reading of an access point in the new sample can be obtained by performing a simple weighted average on the respective access point reading across the *N* records. Table 1 depicts the example of original records (#RSSI) from 1 to n and generated data using Dirichlet data augmentation in A1 and A2 (shaded grey).
**Algorithm 1** Data Augmentation using Dirichlet Distribution (DA-Dirichlet)**Initialise** new_data = [0 for i in range of AP size ’t’]**Initialise** weight ← Dirichlet distribution with α and output size =1**for** *t* = 1 **to** *T* **do**                {for each access_point ’t’}    ** for** *n* = 1 **to** *N* **do**             {for each reference_point ’n’}         new_data[t] += reference_point[n,t] × weight[n]**Result:** new_data

### 2.5. Transfer Learning with WGAN-GP

Conventional machine learning and deep learning algorithms have been traditionally designed to work in isolation. These algorithms are trained to solve specific tasks. The models have to be rebuilt from scratch once the feature-space distribution changes. Hence, transfer learning by feature extraction or fine-tuning from a pre-trained network has been widely used in deep learning applications for adaption of transferable domains, especially for discriminative models. In particular, the WGAN-GP architecture of GANs’ variations has been experimentally demonstrated to be stable and robust for the domain transfer task in the field of computer vision [34].

Utilising the prior obtained knowledge from an organically trained model at the location with the most datapoints ($d_{max}$), as well as shortening the convergence time, we propose to perform transfer learning from $loc_{max}$ (source domain) to $loc_{i}$ (target domain) in this framework using WGAN-GP architecture (explained in Section 2.3 and Table 3. In other words, the saved model state of the WGAN-GP’s generator and critic were used for the transfer learning task by fine-tuning. This method was beneficial for the unique coordinates that contained fewer samples, as it improved the generated image. Since the images were normalised before training, the generated images must be denormalised before saving. The experiment sets the generating image to be the perfect square of the AP numbers, where it requires only 1/4 of originally trained epoch (1000 epoch reduces to 250 epoch) to converge with the transfer learning.

### 2.6. Filtering Module

A filtering module is incorporated to discard any outliers of the generated data from the model. We propose a technique to identify and minimise such data points from being included in the augmented dataset. The proposed technique is robust, unique, simple to implement, and can easily be extended and applied on any dataset. The idea comes from the fact that the outputs generated by the GANs model should be within an allowable deviation range from the corresponding original data samples. Intentionally, the generated image quality assessment, such as Fréchet inception distance (FID) or Inception Score [35], was not used as the measuring matrices in this proposal because they compute the differences between the original and generated data distribution. Because there is a lack of data for certain locations to begin with, the data distribution could not be fairly observed. Instead, in our method, we measure against the maximum allowable threshold Θ.

Given each unique location loc(lat,lon), there exists a sample $\left(RSS_{i},RSS_{j}\right)$ combination where i≠j. Each sample refers to the received signal strength $RSS_{i}=\left\{RSS_{i,1},RSS_{i,2},...,RSS_{i,AP}\right\}$ where AP is the total number of detected access points. The differences between the two vectors ($R\vec{S}S_{i},R\vec{S}S_{j}$) can be computed by summation of absolute differences of the two vectors at each access point ap. Empirically, we found that the L1 norm, the sum of the absolute values of the vector, and the L2 norm yield comparable results in this case; thus, the L1 norm was used to compute the difference. The maximum allowable threshold (Θ) in Equation (5) is the average of maximum of all location’s L1-norm summation of the per-location original data pairs over num_loc, the total number of unique locations present in the dataset. In order to ensure the quality of the generated dataset, the output generated by WGAN-GP at loc ($RSS_{G_{i\in loc}}$) is validated such that the minimum absolute difference (L1-norm) between the generated data and all original data of the location loc is within the allowance of (Θ). Equation (6) depicts the dissimilarity that assesses how identical the augmented fingerprint is to the original one. The lower the score, the more identical the augmented data are to the original ones. It is to be noted that $RSS_{O_{i}}$ and $RSS_{G_{i}}$ are the $RSS_{i}$ of the **O**riginal and **G**enerated dataset, respectively. In summary, the filtering condition is to be confirmed in Equation (7).

(5) $$\Theta =\frac{\max_{(O_{i},O_{j})\in loc}\sum_{ap\in AP}\left|RS{\vec{S}}_{O_{i},ap}-RS{\vec{S}}_{O_{j},ap}\right|}{num\_loc}$$

(6) $$dissimilarity\left(G_{i\in loc}\right)=\min_{O\in loc}\sum_{ap\in AP}\left|RS{\vec{S}}_{G,ap}-RS{\vec{S}}_{O,ap}\right|$$

(7) $$dissimilarity(G_{i\in loc})\leq \Theta$$

## 3. Experiment Design and Data Preparation

The experiments are designed to address two main discussion points:

**E1** 
To verify the effectiveness of the proposed framework (extendGAN+). It is to have in-depth discussion on the generated data and the framework’s performance in comparison with the previous study GAN+ [25].
**E2** 
To evaluate the localisation performance with extendGAN+ datasets. The augmented data is inputted to train the localisation models and compared against baseline methods. It is to address the usability of the proposed augmentation method with the off-the-shelf deep learning methods on two case studies: the widely used public dataset UJILoc and a newly collected building complex dataset (explained in Section 3.1).

### 3.1. Base Dataset: Data Preparation

Two datasets are chosen as the base datasets: the data collection UJIndoorLoc [24] and the newly collected building complex dataset. A base dataset contains training (80% of the original training data), validation (20% of the original training data), and test sets. The input data are the RSS which have gone through data cleaning where undetected RSS are being replaced by −110, thereafter normalised to [0,1].

**Figure 2:**
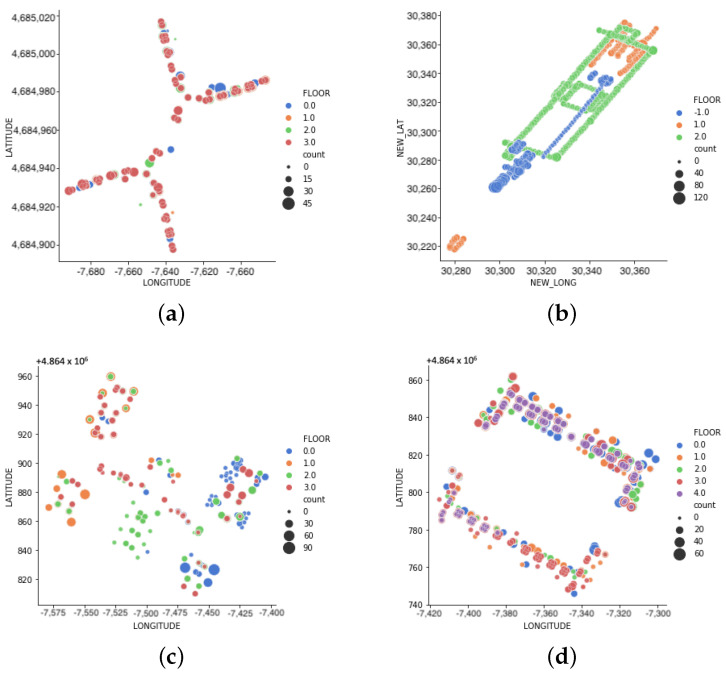
Base Datasets—Data Point Illustration. (**a**) UJI-Building0. (**b**) BC-Building. (**c**) UJI-Building1. (**d**) UJI-Building2.

The UJIndoorLoc (referred as “UJI”) was collected at the Universitat Jaume I, Spain, covering three buildings with four to five floors per building (almost 110.00 m${}^{2}$). It was collected by more than 20 different users and 25 Android devices. The building complex (referred as “BC”) was self-collected at the two-building complex (total area of 46,369 m${}^{2}$) in Singapore. The data collection covered three floors with five participants walk-through on the marked route on two separate occasions/days using Google Pixel 4A, with an in-house data collection application. The details of data collection and preprocessing are presented in Appendix A. Both datasets were selected since they present different challenges in terms of data collection process, data collection layout, and data distribution (Figure 2). While UJI has more data collectors and various devices, BC exhibits a longer stretch of the test bed. Table 2 further illustrates the datasets’ characteristics and parameters. For instance, both UJI and BC have comparable training sizes despite having different building layouts. The data points per location depict the min-max number of records per location to train the localisation model. The numbers of 2 and 4 of UJI and BC respectively are evidently insufficient and highlight the need for data augmentation.

### 3.2. Augmented Dataset: Augmentation Setup

As proposed in Section 2.2, an anchor location was selected for the unique location with the most data points. In this case, $loc_{max}$ was selected with $d_{max}$. The WGAN-GP model was trained with converted-image (23×23 for UJI and 19 for BC) input, with RSS of total APs and padded −110 for the rest. Reducing the training time, for other unique locations, the Dirichlet distribution was used to aggregate the original data points until its data points reach $d_{max}=75$, and the WGAN-GP model was transferred and tuned. In summary, each unique location consists of 150 datapoints (75 real/Dirichlet-aggregated data and 75 WGAN-GP generated data). It is to be noted that the validation and testing sets are not being augmented (i.e., the original data).

Table 3 presents the hyperparameter setting for the experiment. The selection of the hyperparameters was carried out through an iterative process of trial and error, and empirical testing was conducted by observing the Wasserstein distance for the convergence of the model, evaluating the quality of the generated samples by computing the dissimilarity score and testing the efficacy by using it in training with the localisation model. As the transfer learning approach is adopted for the extendGAN+, the training time is reduced to a quarter of the total time as the epoch is reduced from 1000 to 250 for locations other than $loc_{max}$. The training of the localisation model was split between buildings and floors.

### 3.3. Experiment Setup

To study the effectiveness of the data augmentation, localisation methods are needed in order to benchmark against the ground truth. The performance is measured by estimating the root mean square errors (or distance error in meters). Another evaluation metric is measuring the improvement (Improvement%) of the method ($RMSE_{algo}$) against the baseline ($RMSE_{bl}$) are calculated as shown in Equation (8).

(8) $$Improvement\%=\frac{RMSE_{algo}}{RMSE_{bl}}\times 100$$

In this study, the localisation models used for comparison and its configuration (Table 4) are as the following:

- Fingerprint (**FP**): a baseline representing a widely used method deployed in the real environment.
- RandomForest (**RF**): a baseline for the conventional machine learning methods. It is well-suited for small datasets.
- Deep Neural Network (**DNN**): a simple DNN model was trained to predict the user’s location. It is used as a baseline method where the input data are a single sample of RSS data.
- Residual Network (**ResNet**) [36]: a state-of-the-art CNN-based model representation for comparison. In particular, ResNet18 was selected due to its lower number of parameters, as it would take a shorter time to train, and comparable performance with its peers such as MobileNet.
- Residual Network + DNN (**ResNetNN**): a combination of ResNet and DNN such that three fully-connected layers were added to learn the location estimates.
- Time-series-input CNN (**tCNN**) [15]: a CNN-based model that uses consecutive time-dependent RSS as input. Differing from the original paper which stated that the area was grouped into 3 m × 3 m grids in order to produce the [t,AP] input for the model where t=10, each unique location was used instead of having to group the area into the above mentioned grid as sufficient data are created using the augmentation method.

The various hyper-parameters across all models are kept constant. The learning rate of 0.0001 along with an ADAM optimizer was used. The model was trained for 500 epochs with early stopping condition. Preprocessing step converted all RSS values to be between [0,1], where 1 indicates strong signal strength.

## 4. Experiment Results and Analysis

### 4.1. E1: Synthetic Data Quality and extendGAN+ Effectiveness

In order to study the effectiveness of the proposed method, extendGAN+ is being compared with the previously studied GAN+ [25] with two evaluation criterias: data quality and distance error (improvement). It is to be noted that other comparisons, such as the data aggregation methods, are not included due to the fact that GAN+ has been compared with, and concluded to outperform, the afore-mentioned cases.

**Figure 3:**
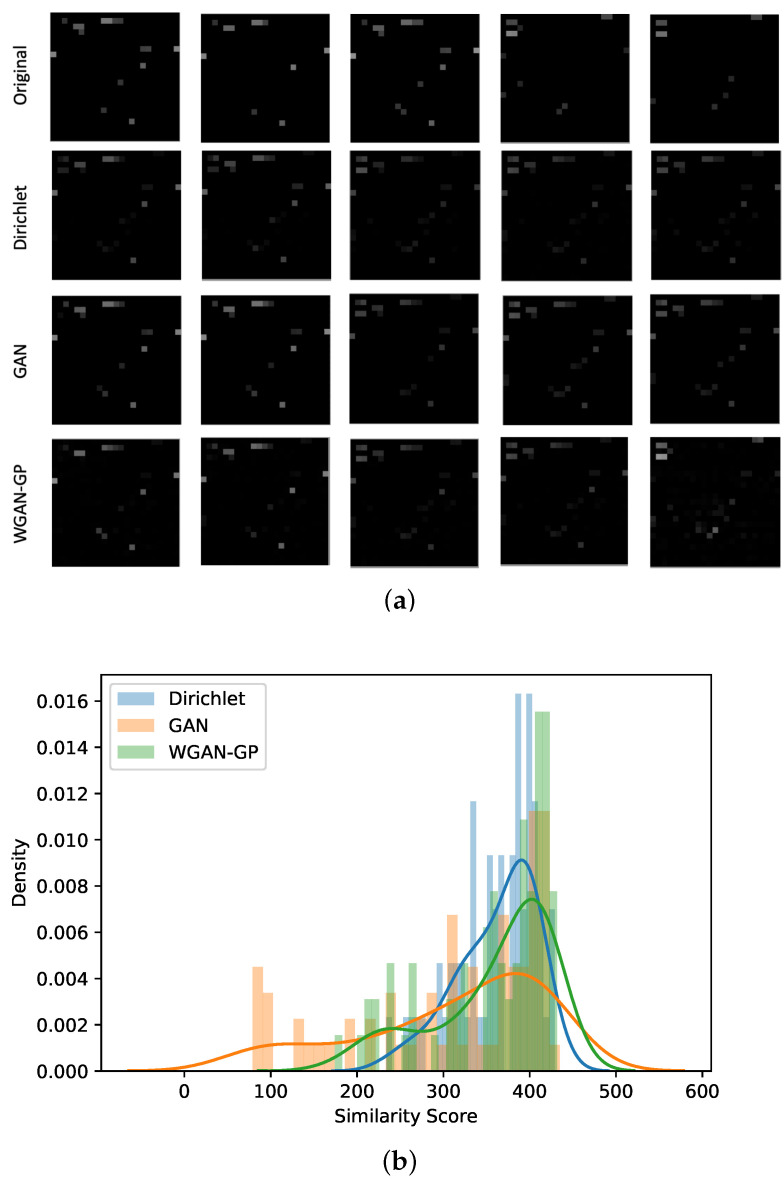
Data Augmentation Quality Example (B0F0, −7637.2570, 4,864,949.8143). (**a**) Data Quality Visualisation—RSS Patterns. (**b**) Dissimilarity Score Density.

Figure 3 shows that the differences between the data augmentation methods discussed in this paper. The case presented is randomly selected from the UJI dataset at Building0, Floor0, and Coordinate [−7637.2570, 4,864,949.8143] with nine original data points. Using the proposed method, we generated 66 Dirichlet, 75 GAN, and 75 WGAN-GP points.

Figure 3a illustrates the variety of the received signal strength (RSS) at a particular location for each AP, where its RSSs are represented in each cells (ranging from black −110 and white 0). Among the nine data points, there are about five unique patterns representing the [−7637.2570, 4,864,949.8143] coordinate. This scenario explains the fingeprint being collected at different occasions at the same place. Hence, the objective of the data augmentation is to be able to replicate this variety of RSS with reduced manual data collection effort. Observing the data produced by Dirichlet, it was as if the five unique AP patterns were combined into one, where the augmented data embodies the interpolation of existing RSS values. Likewise, the data generated by GAN shows similarity to the data of Dirichlet with a few additional patterns. WGAN-GP is observed to produce most versatile behaviour, which almost showcases the unique patterns shown by the original. Moreover, it illustrates new variations with detected AP involved. According to the aforementioned objective, the data from WGAN-GP manage to produce the data as intended.

To quantify the discussion of the synthetic data quality, a dissimilarity score is calculated and Figure 3b presents the probability density distribution of the dissimilarity score of the generated data to the original data. The score is estimated using the minimum absolute difference between the generated data to all original case (explained in Section 2.6 and Equation (6)). It assesses how identical the augmented fingerprint is to the original ones. The histogram represents the actual probability density distribution while the line graph depicts the kernel density estimate (KDE) plot visualising the continuous probability density of the dissimilarity score. The KDE plots shows that Dirichlet data aggregation has the most narrow spread. In fact, the dissimilarity score distribution of Dirichlet is between 233.8 and 428.78 as compared to [78.94, 434.82] and [174.27, 431.80] of GAN and WGAN-GP respectively. It conforms with the prior observation that Dirichlet replicates the data by assigning values to all known APs for the location, hence generating similar sets of data. The results of GAN and WGAN-GP differ in terms of the spread and peak of the dissimilarity score distribution. The dissimilarity score distribution for GAN has a wide range of values. A dissimilarity score of 0 indicates that the generated sample is identical to the original data in both pattern and value. While this suggests that GAN is capable of producing samples that are nearly identical to the original data, the goal of creating synthetic fingerprint data is to generate samples that are similar in pattern to the original data, but different enough to mimic real-world variations in RSS caused by environmental factors such as obstructions or changes in the number of people in the area. Therefore, we desire output that is more dissimilar to the original data, in order to better capture the fluctuations in RSS that occur in the real world. In contrast, WGAN-GP produced a left-skewed distribution with a moderate spread, falling somewhere between the distributions produced by Dirichlet and GAN. This distribution better aligns with the desired characteristics of similarity and dissimilarity of the RSS patterns for the generated data. In comparison to the other methods, WGAN-GP is more suited to generating samples that capture the fluctuation in RSS that occurs in the real-world scenario.

In another aspect, localisation performance could be used to study the effectiveness of its input data. Hence, the average improvement% over GAN+ (Table 5) was derived from the distance error reported in the Appendix B using Equation (8).

Comparing the datasets, it shows that the BC benefits from the extendGAN+ more than from the UJI by showing positive improvement across all localisation methods. The results align with their respective base data’s characteristics such that improvement is more apparent for the dataset that lacks versatility in its base data. In other words, the increase in BC performance is due to its lack of data collection, with only two occasions, five users, and one device type, while UJI may not require much versatility in its pattern as the data were already collected with more devices and users. Regarding the localisation models, ResNetNN with extendGAN+ improves its performance across the building samples, up to 10.13% of improvement, while other methods shows mixed results. The DNN, ResNet and ResNetNN results could be explained such that the deeper and wider the architecture of the model, the more data it may need. Having the deeper structure, ResNetNN benefits from more balanced and versatile data. However, DNN’s, ResNet’s, and ResNetNN’s overall performances have gained better results from the extendGAN+. The poor results of tCNN are not uniquely poor for extendGAN+ but overall for the UJI dataset (shown in Appendix B).

In summary, combining the case illustration and dissimilarity score distribution, WGAN-GP produces more versatile data than GAN and Dirichlet while staying on course with the original data presentation. Thus, the proposed method has shown its effectiveness in generating the variety of RSS cases. The improvement% over GAN+ has reaffirmed the case study by showing that the performance of extendGAN+ improves over GAN+ overall.

### 4.2. E2: Localisation Performance

**Figure 4:**
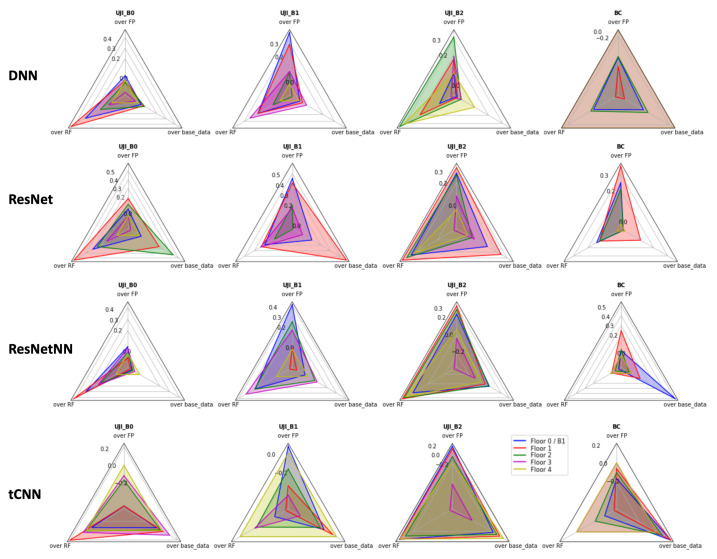
Localisation Performance—extendGAN+ Improvement (improvement%/100).

In this section, the data augmentation, extendGAN+, is evaluated based on localisation performance benchmarks (DNN, ResNet, ResNetNN and tCNN) against baseline methods (FP and RF) and its own base dataset (base_data). The performance benchmark is measured in form of distance error (meter) (Appendix B) and derived fraction of improvement shown in Figure 4.

The overall trend shows the extendGAN+ with DNN, ResNet, and ResNetNN improves the performance over the baseline methods of FP and RF for both datasets, except tCNN for UJI dataset. Over baseline methods, the maximum improvements have been achieved by 23.47% over FP and 25.35% over RF on average. On the other hand, as mentioned in Section 4.1, tCNN performs worse than any others in all categories regardless of data augmentations.

**Figure 5:**
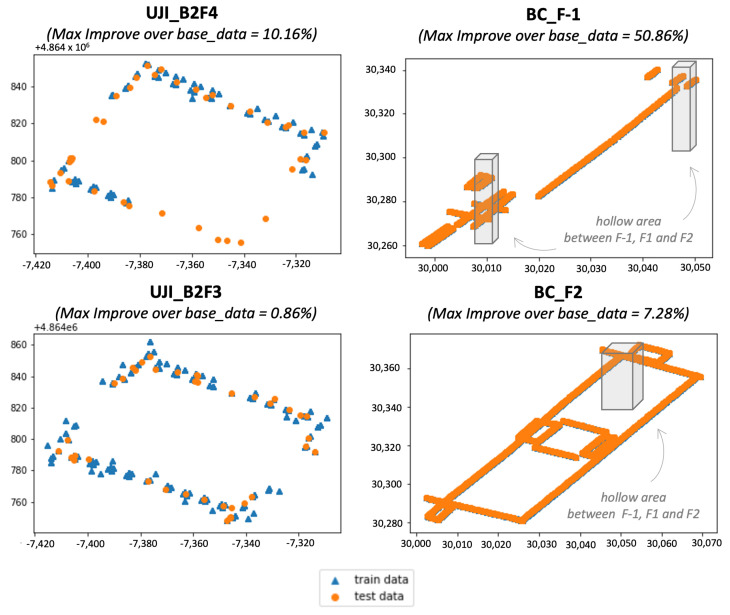
Case study—effectiveness of extendGAN+.

Another important evaluation parameter is “over base_data” where the focus is whether extendGAN+ data augmentation again increases in performance in comparison to the base dataset using the same localisation method. It is not surprising that not all three localisation methods improve at the same rate with the data augmentation. The performance varies depending on the samples and model architecture. The maximum improvement is 18.88% on average. Figure 5 depicts the corner examples of the data augmentation using extendGAN+. The proposed method yields significant improvement at UJI_B2F4 and BC_F-1 in contrast to UJI_B2F3 and BC_F2. UJI_B2F4 does not have the training data covering the test dataset that causes the localisation model to be less accurate with the limited data. BC_F-1 brings up a comparable example to the urban building complex, e.g., a shopping mall, where the center of the area is hollow. In this case, the RSS of APs from other floors are affecting the collected data of the target floor. It causes the prediction to be inaccurate. The data augmentation could add to the fingerprint cases to improve the accuracy.

In this experiment, it could be concluded that the extendGAN+ data augmentation is indeed effective and usable for improving the localisation performance in comparison to the baseline methods and base datasets. It is observed that the extendGAN+ is the most effective when the original data is scarce, incomplete or affected by uncertainty. On the other hands, it is the least effective when the data is sufficient or the area are fully covered by the data collection.

## 5. Conclusions

In this study, we introduced the extendGAN+ data augmentation transferable framework to synthesise RSS data and improve the localisation performance. The recommended framework not only highlighted the effectiveness of using Wasserstein loss and gradient norm penalty to improve the data quality, but also provided a practical guideline for the amount of generated data. The performance of the data augmentation was discussed in two main discussion points, (1) evaluating the data quality and (2) its impact on the off-the-shelf localisation model in comparison to the baseline methods and base datasets. The experiment was carried out on public and self-collected data to address various challenges in data collection and localisation model deployment in the real-world environment. The data quality and dissimilarity score have shown that extendGAN+ is able to produce various RSS pattern and gain improvement up to 10.13% over the previous study GAN+ using ResNetNN model. For the localisation performance, extendGAN+ is is most effective when the orginal data are scarce, incomplete, or prone to mixed signals due to a hollow area in the buildings. It achieves the maximum improvement: up to 23.47% over Fingerprint, 25.35% over Random Forest, and 18.88% over its base dataset. Nonetheless, the limitation of this method boiled down to the combination of the augmented data and its localisation models, as it has been shown in the study that a deeper and wider model favors the data with augmentation as well as the need for versatility in the originally collected data. In future work, we aim to further study the combination of localisation models with the data augmentations, especially localisation models that adopt a federated learning framework against data augmentation.

## Figures and Tables

**Table 1:** Example Augmented Data of DA-Dirichlet.

RSSI	WAP_1	WAP_2	WAP_3	WAP_4	WAP_5	WAP_6	…	WAP_T
1	−110.00	−110.00	−110.00	−110.00	−110.00	−87.57	**…**	−110.00
2	−110.00	−110.00	−110.00	−110.00	−110.00	−88.00	**…**	−110.00
3	−110.00	−56.08	−110.00	−110.00	−110.00	−95.33	**…**	−75.92
4	−110.00	−61.25	−110.00	−110.00	−110.00	−110.00	**…**	−78.94
.								
N	−110.00	−110.00	−110.00	−110.00	−88.00	−87.57	**…**	−110.00
**Augmented Data**								
A1	−110.00	−77.65	−110.00	−110.00	−106.98	−97.92	**…**	−90.16
A2	−110.00	−84.12	−110.00	−110.00	−106.12	−94.90	**…**	−93.61

**Table 2:** Base Datasets Description.

Information	UJI Description	BC Description
Area of data collection	3 buildings, 4–5 floors/building	1 building, 3 floors
Input data	520 access points, RSS range = [−104, 0] or −110 if undetected	345 access points, RSS range = [−110, 0] or −110 if undetected
Label data	Longitude, Latitude (EPSG:3857)	Longitude, Latitude (EPSG:3414)
Training/testing size	19,937/1111	16,157/6465
Per-location data points	[2, 80]	[4, 104]

**Table 3:** extendGAN+ Hyperparameters.

Hyperparameters	GAN+		extendGAN+	
	UJI	BC	UJI	BC
Data (per unique location)	RSS (520)	RSS (345)	RSS image (23 × 23)	RSS image (19 × 19)
Generator	10 (input—latent noise), 128, 256, 512, 1024, 520 (output)	10 (input—latent noise), 128, 256, 512, 345 (output)	100 (input—latent noise), 3 × 3 × 512, 6 × 6 × 256, 12 × 12 × 128, 23 × 23 × 1 (output)	100 (input—latent noise), 3 × 3 × 512, 5 × 5 × 256, 10 × 10 × 128, 19 × 19 × 1 (output)
Discriminator/Critics	520 (input), 512, 256, 128, 1 (output—real/fake)	345 (input), 256, 128, 1 (output—real/fake)	23 × 23 × 1 (input), 12 × 12 × 128, 6 × 6 × 256, 3 × 3 × 512, 1 (output—score)	19 × 19 × 1 (input), 10 × 10 × 128, 5 × 5 × 256, 3 × 3 × 512, 1 (output—score)
Critic Iteration	-	-	5	5
Gradient Penalty	-	-	10	10
Output	RSS (520)	RSS (345)	RSS (23 × 23)	RSS (19 × 19)
Batchsize	4	4	4	4
Epoch	1000	1000	1000 (for $loc_{max}$) 250 (for $loc_{i}$)	1000 (for $loc_{max}$) 250 (for $loc_{i}$)
Learning Rate	0.001	0.001	0.001	0.001

**Table 4:** Experiment Cases.

Localisation Method	Inputs	Convolutional Layers	Hidden Layers	
			UJI	BC
FP	base	-	-	-
RF	base	-	-	-
DNN	base	-	500, 500, 500	300, 300, 300
	GAN+			
	extendGAN+			
ResNet	base	ResNet18	-	-
	GAN+			
	extendGAN+			
ResNetNN	base	ResNet18	500, 500, 500	300, 300, 300
	GAN+			
	extendGAN+			
tCNN	base	Layer1: 8 out channels and 10 × 3 kernel Layer2: 4 out channels and 5 × 3 kernel Pooling: 2 × 2 (stride = 2)	128, 128, 128	128, 128, 128
	GAN+			
	extendGAN+			

**Table 5:** Improvement% over GAN+.

Localisation Methods	UJI			
	B0	B1	B2	BC
DNN	4.87%	−1.24%	3.32%	9.04%
ResNet	−6.00%	2.91%	−2.36%	2.74%
ResNetNN	1.26%	10.13%	6.13%	3.29%
tCNN	2.89%	−15.82%	−15.63%	4.00%

**Figure A1:**
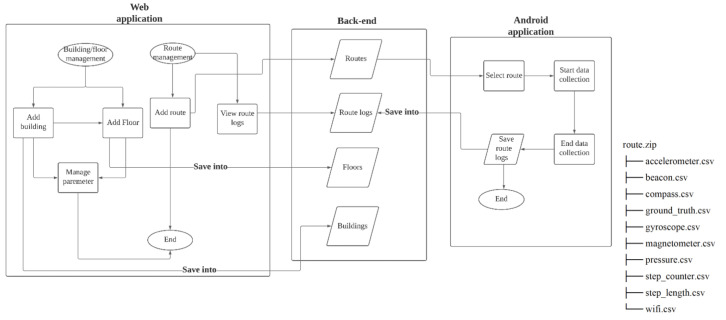
Data Collection Application.

## Data Availability

Not applicable.

## Appendix A. Building Complex (BC)—Data Collection and Pre-Processing

The data are collected at a two-building complex and the experiment is conducted on three floors, over an area of 46,369 square meters. The data collection was carried out by our in-house data collection application and five participants walked along the marked route on two separate occasions/days using Google Pixel 4A.

The raw data were collected using the self-implemented web server and mobile application (Figure A1). Before commencing collecting data on each route, a calibration step was taken place to estimate the user stride’s length, which would aid the ground truth generation.

Subsequently, the data collected are pre-processed as follows:

- Convert the GPS/EPSG:4326 coordinate system (native to the mobile application) to SVY21/EPSG:3414 (meter-based projected coordinate system)
- Filter removable Access Point MAC address:
  - non-stationary MAC address: remove MAC address that are uncommon between the two different data collection session.
  - possible mobile hotspot: remove MAC addresses that appear strong in more than one cluster. It means removing any access-point with a size larger than 26 m-by-26 m.
- Group raw reference points into 2 m-by-2 m grid. Due to individuals having different stride length, the raw data result in too many unique coordinates containing very few sample/data-points.

## Appendix B. Localisation Performance—Distance Error

**Table A1:** Distance Error between Predicted Location and Ground Truth (meters).

Method	Inputs	UJI			BC
		B0	B1	B2	Building
		**Floor 0/Floor B1**			
FP	base	6.701	20.481	13.066	3.103
RF	base	9.008	15.631	12.607	2.302
DNN	base	6.624	12.820	11.597	2.406
	GAN+	6.929	13.106	10.845	8.549
	extendGAN+	6.418	12.753	11.909	6.396
ResNet	base	6.351	13.959	10.618	2.278
	GAN+	5.760	10.502	9.969	2.203
	extendGAN+	6.213	11.101	9.437	2.023
ResNetNN	base	5.829	11.782	10.775	2.565
	GAN+	6.538	12.904	11.687	2.444
	extendGAN+	6.301	12.160	10.187	2.318
tCNN	base	9.049	19.160	9.532	3.961
	GAN+	9.443	18.260	8.872	3.534
	extendGAN+	9.621	21.904	11.030	3.303
		**Floor 1**			
FP	base	5.623	13.626	9.260	3.919
RF	base	10.172	11.837	9.434	3.521
DNN	base	5.967	9.962	7.479	3.671
	GAN+	6.146	9.429	8.304	6.711
	extendGAN+	5.631	9.675	7.617	6.949
ResNet	base	6.095	18.502	8.261	3.072
	GAN+	4.739	10.138	6.550	3.143
	extendGAN+	4.556	7.977	6.264	3.104
ResNetNN	base	5.327	12.052	6.415	3.606
	GAN+	5.993	15.470	6.542	3.550
	extendGAN+	5.835	13.496	6.388	3.702
tCNN	base	8.217	17.373	8.276	4.642
	GAN+	7.752	18.062	9.289	4.312
	extendGAN+	8.077	18.081	8.317	4.304
		**Floor 2**			
FP	base	5.591	13.138	12.843	2.991
RF	base	6.524	12.772	13.387	2.598
DNN	base	6.133	11.360	8.898	2.627
	GAN+	5.994	11.517	9.713	5.663
	extendGAN+	5.713	12.042	8.853	5.351
ResNet	base	8.617	10.406	9.142	2.213
	GAN+	4.508	9.675	8.539	2.210
	extendGAN+	4.884	10.352	9.468	2.237
ResNetNN	base	5.188	10.589	9.815	5.767
	GAN+	5.054	10.957	12.019	3.113
	extendGAN+	5.545	9.835	9.380	2.834
tCNN	base	6.386	12.821	12.661	4.198
	GAN+	6.855	12.425	11.685	3.657
	extendGAN+	6.481	15.231	13.577	3.465
		**Floor 3**			
FP	base	6.499	9.502	11.091	-
RF	base	7.501	11.551	8.750	-
DNN	base	7.021	9.206	9.040	-
	GAN+	7.172	8.588	8.639	-
	extendGAN+	7.239	8.630	8.964	-
ResNet	base	5.857	8.602	10.012	-
	GAN+	5.838	7.924	9.287	-
	extendGAN+	6.518	7.685	10.139	-
ResNetNN	base	5.889	8.697	10.293	-
	GAN+	6.764	8.906	10.272	-
	extendGAN+	6.190	7.860	11.509	-
tCNN	base	8.008	9.520	10.871	-
	GAN+	8.220	11.195	10.929	-
	extendGAN+	7.219	13.504	17.889	-
		**Floor 4**			
FP	base	-	-	15.461	-
RF	base	-	-	20.247	-
DNN	base	-	-	15.626	-
	GAN+	-	-	16.149	-
	extendGAN+	-	-	14.037	-
ResNet	base	-	-	14.702	-
	GAN+	-	-	15.618	-
	extendGAN+	-	-	15.845	-
ResNetNN	base	-	-	13.959	-
	GAN+	-	-	16.583	-
	extendGAN+	-	-	14.554	-
tCNN	base	-	-	19.683	-
	GAN+	-	-	19.362	-
	extendGAN+	-	-	18.029	-
